# Understanding and preventing nonadherence and treatment dropout in adolescents and young adults with anxiety and depressive disorders

**DOI:** 10.3389/fpsyt.2023.1174285

**Published:** 2023-11-22

**Authors:** Ruth C. Waumans, Anna D. T. Muntingh, Neeltje M. Batelaan, Anton J. L. M. van Balkom

**Affiliations:** ^1^GGZ inGeest Specialized Mental Health Care, Amsterdam, Netherlands; ^2^Department of Psychiatry, Amsterdam UMC, Location Vrije Universiteit, Amsterdam, Netherlands; ^3^Amsterdam Public Health Institute, Amsterdam, Netherlands

**Keywords:** adolescent, young adult, dropout, nonadherence, anxiety disorder, depressive disorder

## Abstract

Dropout from psychological or pharmacological treatment for anxiety and depressive disorders is common. It is especially problematic in adolescents and young adults because of the adverse consequences for their development. Reasons for treatment dropout can be divided into therapy-process related factors, attitudinal aspects, and practical issues. Adjusting treatment to patient preferences and shared decision making, improving the therapeutic alliance, and interventions such as (family) psychoeducation, motivational interviewing, and help with practical issues are promising strategies to optimize engagement and adherence.

## Introduction

Anxiety and depressive disorders are prevalent among adolescents and young adults, with 12-month prevalences of 6%–9% for depressive and 18%–25% for anxiety disorders (1–3). In young individuals the occurrence of these disorders is not only related to increased suicidality and comorbidity but may also lead to problems critical to this life phase such as poor academic performance and troubled social functioning (4–6). In addition, it may entail mental disorders and worse physical health in adulthood (7, 8).

To limit and prevent these adverse consequences, providing adequate treatment is of utmost importance, yet many adolescents and young adults drop out of treatment (9–11). Meta-analyses on dropout from randomized controlled trials (RCTs) regarding psychological and pharmacological treatment for adolescent depression reported overall dropout rates of 14% and 23%, respectively (9, 10), with even higher rates (33%) in the subgroup of older adolescents (>16 years) (9). This figure is probably an underestimation of actual dropout in clinical practice (12). Besides dropout, nonadherence to treatment may undermine the effectiveness of treatment in this age group.

To improve adherence, it is essential to understand why young individuals suboptimally adhere to or even discontinue treatment and which prevention strategies are beneficial.

This Perspective article discusses reasons for nonadherence to and dropout from treatment for depressive and anxiety disorders in adolescents and young adults. In addition, it shows that several strategies are available to prevent these suboptimal treatment outcomes. We also provide suggestions for further research with the aim to improve treatment trajectories for this vulnerable age group.

## Factors related to nonadherence to and dropout from treatment

Factors that appear related to treatment dropout can be classified in three categories.

### Therapy-process related factors

To investigate types of dropout from psychotherapy, O’Keeffe and colleagues (13) performed mixed methods research (including ideal type analysis). They interviewed depressed adolescents aged 11–17 years who discontinued psychotherapy, and their therapists, leading to the identification of three categories of non-completers. A first group was defined as ‘got-what-they-needed’, meaning these adolescents abandoned therapy because they found they had improved sufficiently. In this group, therapists appeared not worried about the therapy discontinuation of their patients and this group had better outcomes than other types of non-completers at post-treatment (nine months). In line with these findings, an older study among students receiving counselling (diagnoses not reported) (14) found that the absence of a perceived need for (further) treatment was an important reason for dropout. A second group in O’Keeffe’s study was termed ‘dissatisfied’ dropouts (13), containing adolescents who were discontent with the therapy offered. Among the issues raised by this group were not finding the therapy beneficial or being dissatisfied with the therapeutic alliance. Interestingly, the therapists of the dissatisfied group seemed unaware of the patients’ opinions and critique about therapy. In a subsequent study by O’Keeffe and colleagues using therapy recordings (15), this group appeared to experience a poorer patient-therapist relationship with more (unresolved) alliance disruptions compared to completers and got-what-they-need dropouts (15). It was found that signs of poor alliance were often already noticeable early in psychotherapy. A third group, the ‘troubled’ dropouts could not adhere to therapy because of insufficient stability in their living situation (see below ‘Practical issues’).

Concerning pharmacotherapy, factors related specifically to the prescribed medication seem to have a large impact on dropout. A meta-analysis of RCTs in depressed adolescents revealed adverse side effects as a major reason for non-adherence (9). Rohden and colleagues performed a qualitative analysis on side effects that were mentioned in the included RCTs, and were related to dropout. They found that adverse events such as attempted suicide, mania, skin rash, and headache were related to dropout (9). Alarmingly, other research indicates that serious adverse events (i.e., suicidal ideation or behavior) among adolescents and young adults are insufficiently monitored in clinical practice (16). Apart from side effects, type of antidepressant also appeared to influence non-adherence: SNRIs were associated with the highest treatment dropout (45%), compared to SSRIs and TCAs (21 and 28% respectively) (9). Correct dosing of antidepressants has been found to positively influence adherence in depressed youth (17). Furthermore, lower dropout rates were found in treatment consisting of a combination of medication and cognitive-behavioral therapy (CBT) compared to medication alone (9).

### Attitudinal aspects

Attitudinal aspects play a role in adherence and treatment engagement. In a qualitative study interviewing primary care providers on barriers to treatment for depressed adolescents, the important influence of parental attitudes was highlighted. Primary care providers mentioned that parents’ negative attitudes towards care impeded treatment access, while parental support facilitated engagement, e.g., through monitoring of medication side effects or enabling attendance of appointments (18).

Results from other populations (including youngsters with a variety of psychiatric diagnoses) may add to these findings. For pharmacotherapy specifically, it was found that those adolescents whose family was less supportive or less knowledgeable about their situation, appeared less adherent (19). In addition to parental attitudinal factors, perceptions of treatment and (limited) motivation for change of youngsters themselves also play a role according to a study among clinicians who treat adolescents with a variety of mental health problems (20). The clinicians in this study reported that adolescents’ positive beliefs about treatment and motivation increased adherence. On the contrary, negative attitudes towards self and treatment were reported as a barrier by the clinicians (20). Indeed, attitude towards professional help has been found to influence adherence to appointments in a cohort of adolescents with affective disorders (21). Attitudinal aspects such as wanting to deal with problems alone or a need for autonomy have been found to play a role in treatment-seeking in adolescents and young adults (22, 23). Autonomy was also an important factor in medication usage according to a narrative review of qualitative studies on psychotropic medication utilization among young individuals (13–24 years), and (perceived) lack of autonomy may lead to nonadherence (24).

### Practical issues

Lastly, practical barriers related to (non) adherence have been reported.

Regarding depressed adolescents specifically, O’Keeffe and colleagues identified a group of adolescents who dropped out because of psychosocial problems, e.g., housing problems, caring obligations, or financial issues (13). Therapists and patients who participated in this study reported similar reasons for discontinuation, and therapists thought regaining stability in life had to be prioritized. A study on factors related to treatment dropout in adolescents and young adults (aged 12–21 years) following psychodynamic treatment for various diagnoses also found that amongst other factors, living situation (i.e., being homeless) was related to dropout (25). Being homeless was found to negatively impact on medication adherence in young individuals with various diagnoses as well (24).

In a sample of (primarily) anxious and depressed students, forgetting to collect the prescription has been identified as an important barrier to adequate medication use (26). In addition, limited opening hours of the pharmacy appeared hindering; as well as being uninformed that medication was prescribed by the physician (26).

Practical issues such as lack of time, scheduling problems and forgetting the appointment were also reported by the previously mentioned studies (14, 20) as barriers to adherence.

## Strategies to prevent nonadherence to and dropout from treatment

Several strategies seem applicable to improve adherence and these will be discussed below.

### Applying shared decision making and adapting treatment to patient preferences

There are indications from studies among adolescents and young individuals with (previous) depression (27) and a variety of mental diagnoses (24) that lack of involvement and/or perceived lack of autonomy may be related to (medication) nonadherence. Therefore, McMillan and colleagues conclude that involving young patients in shared decision making should be enhanced (24).

How and to what extent do youngsters want to be involved? Qualitative research in a small sample of young (previously) depressed individuals (aged 12–24) and their caregivers (27) showed that young patients and their caregivers like to be involved in decision making at least to some degree and most have a wish for more information about treatment. The wish for participation varied across individuals and over time. All youngsters preferred a collaborative form of decision making with at least some involvement from the professional.

Goal setting can be considered to be a shared decision-making technique and was investigated in a recent literature review (28). It was found that setting goals together with the youngster may enhance collaboration and communication, may positively affect the therapeutic alliance, and may increase feelings of being supported and in control.

There seem to be no studies investigating the effect of aligning treatment with patient preferences on dropout in adolescents and young adults specifically. However, several meta-analyses in adults have indicated that adapting therapy to patient preferences reduces dropout (29–31). Moreover, results from our study on treatment preferences of adolescents and young adults with depressive symptoms indicated that providing therapy in accordance with patient preferences would considerably increase willingness to engage (32). For young individuals, specifically those who are ambivalent about treatment, this seems delicate, as for certain unpreferred but common treatment scenarios predicted uptake was found to be negligible (i.e., 3%) (32). Participants stated a preference for individual therapy and treatment with high effectiveness, short waiting time, frequent contacts, and evaluation of the therapeutic bond early in therapy. Interestingly, subgroups could be identified with different preferences for treatment.

While this study was exploratory in nature, Simmons and colleagues (33) designed and evaluated an online decision tool to help depressed adolescents articulate their preferred treatment. They found positive effects on decision making and satisfaction, a decrease in depressive symptoms, and a high treatment adherence rate of 88%.

### Psychoeducation interventions

As described above, youngsters’ attitudes and parental support seem to have an impact on treatment dropout. Hence, paying attention to or even altering these attitudes might be beneficial. Although research on psychoeducation interventions in young individuals with (or at risk for) depression is limited, a systematic review on such interventions suggests positive effects on treatment adherence in adolescents (34). Interestingly, studies on family psychoeducation for adolescents with depressive (35) and mood (36) disorders have shown positive results (35, 36). These include fewer improper assumptions and increased understanding of depression and therapy in parents (36), an improved bond between adolescent and parent and higher parental contentment with therapy (35).

Research in adults has shown beneficial effects of psychoeducation on treatment adherence both in psychotherapy and pharmacotherapy (37, 38). Even a few written sentences (on the dose-effect model of psychotherapy) shared at the start of an intake appointment appeared to yield a 3.5 higher chance to become a psychotherapy completer versus a non-completer (37). Furthermore, specific role induction strategies (i.e., providing information about therapy customs, including the role and behavior of therapist and client) have been found to positively influence dropout in adults (39). These strategies seem worthwhile to try among adolescents and young adults as well.

### Motivational interviewing

Another intervention that focuses on attitudinal aspects is motivational interviewing (MI), and research suggests that MI may positively influence treatment engagement in adolescents (40, 41). A study amongst adolescents with anxiety and mood disorders (40) found that motivational interviewing had a positive effect on adherence to group psychotherapy. Participants individually received a single MI-session or an active control alternative before the start of group therapy. Participants who received MI more frequently started treatment (96% in the MI group vs. 80% in the control group) and had greater adherence to the group sessions. As even one single session of MI appeared beneficial, implementing such an intervention seems highly feasible. MI has also shown to be valuable in enhancing medication adherence in adolescents (41). Thus, motivational interviewing seems a useful intervention for optimizing adherence in adolescents and young adults.

### The therapeutic alliance

As mentioned before, poor therapeutic alliance has been related to treatment dropout in adolescents (13, 42), which is corroborated by more extensive research in adults (43). Hence, assessment of the working alliance should be given the necessary attention. One possible strategy to optimize the therapeutic alliance is a standard evaluation after three sessions to assess the patient-therapist bond, thereby facilitating discussion of the working alliance and potential adjustments. This hypothesized option received positive appraisal in our sample of depressed adolescents and young adults (32). This approach is supported by the finding that missed appointments early in therapy are related to dropout (42) and that therapists may be unaware of dissatisfaction with therapy experienced by their young patients.

How to improve the therapeutic alliance with the patient? A recent meta-analysis provides an overview of beneficial and less beneficial approaches regarding the alliance with youth clients. The meta-analysis included a sample of youngsters (mean age 14 years, both with psychiatric diagnoses, as well as nonclinical individuals) and therapists (44). One of the most important aspects to strengthen the alliance is to emotionally align with the patient; e.g. through exploration and acknowledgement of the patient’s feelings and experiences, as well as by being supportive and general enhancing factors such as being empathic, authentic, respectful and trustworthy. Another aspect can be best described as ‘collaboration’, and comprises the therapist becoming an ally, cooperating with the patient and enhancing a feeling of agreement and equality, and promoting independence. Being too directive or dominant (e.g., forcing to talk) and generally hampering therapeutic factors (e.g., being critical, nonsupportive, nonresponsive or too formal) were found to be unbeneficial for the working alliance.

### Help with practical issues

Since practical issues such as forgetting appointments or medication have been found to negatively influence adherence, we looked for interventions tackling these issues. Findings from one study (45) imply that medication reminders via a cell phone app can increase compliance to antidepressants in college students. Research in a population with somatic disease suggests that linking medication use to daily routines (e.g., brushing teeth or showering) might be another facilitator (46).

## Discussion

This Perspective article discusses reasons for nonadherence and treatment dropout among adolescents and young adults with anxiety and/or depressive disorders and potential strategies to reduce nonadherence and prevent dropout in this group. We found that studies investigating this topic are scarce, indicating the need for more research on this important clinical problem. Specifically, research on the relationship between attitudes of young individuals and dropout is needed to inform development of strategies to prevent dropout. Of all literature included in this article, studies focusing on adolescents and/or depression were overrepresented; the findings may therefore be more applicable to this specific group. The group of young adults, as well as adolescents and young adults with anxiety disorders, deserve particular attention in future studies.

Interpretation and comparison of included studies was compromised because of the use of different definitions of treatment dropout (e.g., “the adolescent ending treatment without the prior agreement of their therapist, regardless of when in treatment the ending occurred” (13) versus “adherent behavior was defined as taking between 80% and 100% of prescribed medication” (45)). Nonetheless, from the available information, the following suggestions for clinical practice can be formulated but should be interpreted with caution.

### Implications for clinical practice

As can be concluded from the aforementioned findings, clinicians should be alert to imminent treatment dropout and be aware of various reasons for nonadherence or dropout. Several strategies at different stages in therapy can be undertaken to increase engagement and adherence. Suggested strategies are summarized in Table 1.

**Table 1:** Suggested strategies for clinical practice.

Time point	Strategy
Before start of treatment	(Family) psychoeducation & role induction
	Motivational interviewing
During (early) treatment	Establish treatment plan in line with patient preferences Evaluate therapy progress and side effects
	Evaluate therapeutic alliance
	Evaluate need for further treatment
	Evaluate need for help with practical issues

#### Before the start of treatment

- Psychoeducation and expectation management seem important. Specifically, education on treatment duration and when to expect therapy effects seems helpful (37), as well as informing that for medication adverse effects may emerge before the intended effect. Engaging family in psychoeducation programs can be beneficial and should be considered (35, 36). Also, role induction can be applied. This can be done by sharing verbal or written information, or by video demonstration (39).
- An intervention with motivational interviewing is recommended to increase motivation and adherence, especially for individuals with hampering attitudes or limited motivation, as this was found beneficial in a sample of adolescents with anxiety and mood disorders (40). Even a single session appears to yield positive effects (40).

#### During (early) treatment

- A treatment plan should be established in line with patient preferences, optionally with the aid of a decision tool (33).
- Regular evaluations during therapy may give insight into whether an adolescent considers quitting and for which specific reason. Topics to assess include the following:

- *Therapeutic alliance and therapy progress*. From the beginning, building a strong connection with the patient should be prioritized, and attention should be paid to formulating matching therapy goals. Signs of poor therapeutic alliance (e.g., alliance disruptions) and early missed appointments should be given particular attention. In pharmacological treatment, physicians should monitor medication side effects, as this is related to treatment dropout. Therapy progress may be monitored with routine outcome monitoring, and rating scales are available for evaluation of the therapeutic alliance.
- *Need for further treatment.* This should be properly examined, using motivational interviewing techniques to explore remaining ambivalence. Concluding treatment when therapy goals are achieved may lead to more concise treatment in some cases, preventing dropout of those with no need for further treatment.
- *Practical issues*. Relevant psychosocial problems and scheduling issues should be given the necessary attention. As forgetting to collect the medication, not knowing that medication was prescribed, as well as being hindered by limited pharmacy opening hours were found to be barriers to medication adherence (26), possibilities to extend the opening hours of pharmacies or mental health institutions could be explored. Furthermore, young individuals should be encouraged to plan medication collection, preferably directly after prescription. Medication reminders via cell phone apps can be considered (45).

### Conclusion

Dropout from and nonadherence to treatment among depressed and anxious young individuals is common and often with negative consequences. Important reasons for dropout can be divided into therapy-process related factors, attitudinal aspects, and practical issues. Therapy-process related factors, such as not experiencing a need for further treatment or contrarily, dissatisfaction with treatment, might be addressed by adjusting treatment to patient preferences and shared decision making, paying attention to the therapeutic alliance and frequent evaluation during therapy. Patient and parental attitudinal aspects require adequate psychoeducation (including role induction) and motivational interviewing interventions. Lastly, practical issues could be tackled by using reminder apps, and by paying specific attention to scheduling of appointments and medication collection, as well as psychosocial problems that interfere with treatment adherence. The field could benefit from more research on attitudinal factors contributing to treatment dropout, as well as research in specific underrepresented samples such as young adults and patients with anxiety disorders.

## Data availability statement

The original contributions presented in the study are referred to in the reference list.

## Author contributions

RW, AM, NB, and AB have contributed to the design of this study. RW has performed the literature search and has performed the primary data analysis of the included literature. AM, NB, and AB have contributed to the interpretation of the data and supervision of the data analysis. The draft manuscript was written by RW and further reviewing, and editing was performed by RW, AM, NB, and AB. All authors contributed to the article and approved the submitted version.
